# Linking individual medicare health claims data with work-life claims and other administrative data

**DOI:** 10.1186/s12889-015-2329-6

**Published:** 2015-09-30

**Authors:** Elizabeth Mokyr Horner, Mark R. Cullen

**Affiliations:** American Institutes for Research, 2800 Campus Drive, Suite 200, San Mateo, CA 94403 USA; Stanford University, Stanford Medical School, MSOB 1265 Welch Rd X338, Stanford, CA 94305 USA

**Keywords:** Linkage, Harmonization, Medicare, Health claims, Social epidemiology

## Abstract

**Background:**

Researchers investigating health outcomes for populations over age 65 can utilize Medicare claims data, but these data include no direct information about individuals’ health prior to age 65 and are not typically linkable to files containing data on exposures and behaviors during their worklives. The current paper is a proof-of-concept, of merging employers’ administrative data and private, employment-based health claims with Medicare data. Characteristics of the linked data, including sensitivity and specificity, are evaluated with an eye toward potential uses of such linked data. This paper uses a sample of former manufacturing workers from an industrial cohort as a test case. The dataset created by this integration could be useful to research in areas such as social epidemiology and occupational health.

**Methods:**

Medicare and employment administrative data were linked for a large cohort of manufacturing workers (employed at some point during 1996–2008) who transitioned onto Medicare between 2001–2009. Data on work-life health, including biometric indicators, were used to predict health at age 65 and to investigate the concordance of employment-based insurance claims with subsequent Medicare insurance claims.

**Results:**

Chronic diseases were found to have relatively high levels of concordance between employment-based private insurance and subsequent Medicare insurance. Information about patient health prior to receipt of Medicare, including biometric indicators, were found to predict health at age 65.

**Conclusions:**

Combining these data allows for evaluation of continuous health trajectories, as well as modeling later-life health as a function of work-life behaviors and exposures. It also provides a potential endpoint for occupational health research. This is the first harmonization of its kind, providing a proof-of-concept. The dataset created by this integration could be useful for research in areas such as social epidemiology and occupational health.

## Background

### Introduction

Over the last decade, research has increasingly utilized administrative claims data to evaluate health outcomes [1–4], providing an insight into population health even without test results or clinician notes [3–6]. Researchers now often have the option to evaluate the impact of work-life and environmental characteristics on health by linking insurance claims from individuals’ work-lives with other administrative databases available on insured employees, such as demographics, job status, environmental exposures, and socioeconomic measures [7–9].

However, data on claims via employment-based insurance are often censored at age 65, or earlier for individuals who leave their workplaces, when Medicare becomes primary for individuals who are not otherwise insured. Researchers investigating health outcomes for populations over age 65 can utilize Medicare claims data [10–13]. These Medicare claims data subject to less selection bias, but include no direct information about individuals’ health prior to age 65, and are not typically linkable to files containing more than superficial demographic, economic, behavioral, or environmental data on the covered individuals.

The current paper provides a proof-of-concept for merging employers’ administrative data and private, employment-based health claims with Medicare data, using a sample of former manufacturing workers from an industrial cohort as a test case. Characteristics of the linked data, including sensitivity and specificity, are evaluated with an eye toward potential uses for such linked data.

Social epidemiologists and health economists could use integrated Medicare and pre-retirement data to answer a variety of questions regarding how health trajectories emerge, utilizing previous health information to explore how individuals’ risks for chronic diseases evolve over time. Further, administrative data on work-life characteristics and behaviors (e.g., tenure, absences, disciplinary actions, etc.) can shed light on how exposures and behaviors change health trajectories. For example, a question of considerable interest to all “payors”—employers and the US government—as well as to the workers themselves is how health trajectories in retirement may be affected by the timing of retirement and the incentives to retire. This data integration would provide researchers with unprecedented insight into how health after age 65 is shaped over the life course.

This paper has several important findings. First, for individuals who are continuously insured, we show how consumption of medical care changes as they transition from generous private insurance to public insurance as their primary coverage. Subsequently, we compare Medicare data with employer-sponsored insurance to explore sensitivity and specificity of these data. Our data reveal how much health history is lost when researchers work solely with Medicare data. Finally, data on health status from individuals’ work-lives are considered. The medical claims trajectory (or *risk score*, a proxy for health trajectory) at age 61 is considered, as are specific biometric data from medical exams obtained at work that are available for a subset of the cohort. We conclude with a summary of lessons for health researchers who may utilize claims data for future studies relating later-life health status to work-life behaviors, exposures, and environmental conditions. The motivations for this linkage are threefold, as discussed below.

### Motivation 1: Linkage allows for evaluation of health outcomes of retirees

A strong international effort has been made to survey individuals as they transition from middle-age into old age (the Health and Retirement Survey (HRS) in the US; the Survey of Health and Retirement in Europe (SHARE); the Chinese Health and Retirement Survey (CHARLS); and many others). Data from these surveys have many strengths, including providing a relatively representative sample and survey questions on subjective experiences that provides insight into quality of life. However, these survey data are prone to specific weaknesses for public health research. Specifically, survey health data have been demonstrated to 1) lack of concordance with medical records [14]; 2) be confounded by mood and affect [15]; and 3) suffer in quality particularly if cognitive decline is possibly present [16] and especially if precise onset dates are necessary [17, 18]. A linkage like the one described here could be used to evaluate individuals’ health trajectories in a more objective way than survey data allows.

### Motivation 2: Linkage provides an outcome or endpoint for research on work-life exposures

When considering the health effects from work-life exposures, using health status from private insurance is problematic. Selection out of employment is unlikely to be random even during mass layoffs [19] and thus, analyses restricted to active workers must always be viewed as survivor studies suffering from healthy worker selection bias [3]. To circumvent this, many researchers have opted to use mortality as the endpoint, using the National Death Index (NDI), which provides information on the timing and causes of death for entire cohorts, regardless of when they may have left employment.

However, NDI data have several major shortcomings when used for this purpose, including: 1. Researchers must wait until the workers die, which may be decades after the exposures of interest; 2. Notable successes at modifying the natural history of common ailments has obscured many causal pathways to cause-of-death; and 3. Focusing on mortality ignores quality of life in the decades of life after retirement. In some ways, whether or not individuals have chronic illnesses during these years may be of more importance to their own welfare—and society’s—than whether they die at 85 or 90 from cardiovascular disease or cancer. Thus, combining administrative data from work-lives with Medicare data may provide an excellent alternative for investigating the effects of exposures or treatments over the life course.

### Motivation 3: Linkage provides insight into what is missed when evaluating a Medicare-only cohort

In addition, this paper provides a window into what researchers using only Medicare data would observe, as compared to what they could have observed if they had access to earlier health claims during work-life. This is especially relevant for the study of disease incidence since claims research rely on earlier diagnoses to establish the denominator, usually through a wash-out period. These data allow us to see how much of health history is captured using a two-year wash-out period.

## Methods

### Conceptual framework

The primary goal of this paper is to demonstrate the integration of Medicare data and other administrative and health data from previous work-life. Because this is a proof-of-concept paper, the analyses are descriptive by design and methods designed for causal inference are unnecessary. The outcome variables considered are a set of chronic diseases (coronary heart disease, asthma, diabetes, hypertension, arthritis). These were chosen because of their relatively high preexisting rates in this working population, which researchers using only Medicare data would not typically be aware of. In addition, these are precisely the types of conditions that impact quality of life but that in many cases can be treated such that data on mortality will not provide a clear insight into what happened between leaving long-term employment and death.

In some cases, major depression is also considered despite its low prevalence in diagnostic codes because it is one of the only ways of measuring mental health using claims data. Other conditions of interest, such as cancer, could not be examined because of their rarity pre-retirement and limits of data availability in this still relatively young cohort all actively working at least as recently as 1996. The goals and empirical framework are described below.

First, to describe the characteristics of the integration, the concordance in diagnoses between private and public insurance is explored. This provides insight into how the integrated data behave and how previous claims can be used to clarify preexisting risk trajectories. Specifically, private (employment-based) and public (Medicare) claims data are compared for consistency. This is the first step to establish the validity of this type of data integration, as it is important to understand what any change in the inferred rates of diagnoses means.

Next, individuals’ behaviors are examined. Specifically, discontinuities in health consumption and diagnoses are investigated in order to determine whether individuals consume more or less medical care when transitioning onto Medicare from relatively generous private insurance.

Data from pre-Medicare life is then used to predict outcomes for individuals. Two primary pre-Medicare individual-level characteristics are considered: risk scores, which are an actuarial forecasting tool used to predict health expenditures for the following year, and biometric data on lung function and body mass index (BMI) that were gathered at the jobsite. These analyses demonstrate how prior information on risk can be informative regarding health later in life. These biometric data were gathered as part of plant-specific occupational health screening efforts. As such, individuals measurements were taken when workers were at different ages.

### Data sources and linkage

Medicare data were linked to health claims from private employer-purchased insurance. Data were obtained for hourly and salaried employees at a geographically diverse aluminum production company who worked a day or longer between January 1, 1996 and December 31, 2009. This company had remarkably complete data, including biometric measures from periodic medical exams. Medicare data were linked using social security numbers submitted to the Center for Medicare and Medicaid Services.

### Sample

Worker characteristics were obtained from personnel records and linked using a commonly scrambled employee ID number. Summary data are presented in Table 1, with samples used demarked with numbers (e.g., Sample 1) while subsamples aimed at describing the data are demarked with letters (e.g., Sample A).

**Table 1:** Sample summary

a: All Data					
	Sample A [SA]	Sample B [SB]	Sample C [SC]	Sample D [SD]	Sample E [SE]
	*Everyone over 65*	*SA + Matched in CMS Data*	*SB + Not HMO in CMS Data*	*SC + Hourly Men*	*SD + Not Outlier*
N	10,571	8,321	6,810	3,886	3,737
Men	78.1 %	84.4 %	84.6 %	*	*
Hourly	57.9 %	66.8 %	66.8 %	*	*
Birth Year [SD]	1939 [3.2]	1940 [3]	1939 [2.9]	1939 [2.7]	1939 [2.7]
Ever Married	64.4 %	67.6 %	67.5 %	71.4 %	72.0 %
Coronary Heart Disease	24.5 %	27.1 %	29.1 %	32.0 %	31.5 %
Hypertension	60.5 %	66.2 %	70.5 %	71.6 %	71.3 %
Diabetes	26.2 %	28.2 %	29.9 %	32.6 %	32.5 %
Asthma	14.1 %	16.0 %	17.5 %	18.6 %	17.8 %
Arthritis	39.4 %	43.6 %	46.4 %	45.4 %	45.3 %
Risk Score	2.21	2.03	2.03	2.04	2.00
b: Samples in Paper					
	Sample 1	Sample 2	Sample 3	Sample 4	Sample 5
	*SE + Plants did not close*	*SE + Continuous data available*	*SE + Risk Score at age 61 available*	*SC + Male + BMI data available*	*SC + Male + Lung function data available*
N	1,711	1,592	1,552	367	1,364
Men	*	*	*	*	*
Hourly	*	*	*	*	*
Birth Year [SD]	1940 [2.2]	1940 [2.2]	1940 [2.1]	1941 [1.7]	1940 [2]
Ever Married	85.6 %	86.4 %	86.3 %	89.1 %	87.8 %
Coronary Heart Disease	32.0 %	32.5 %	33.1 %	28.9 %	26.9 %
Hypertension	75.6 %	76.3 %	74.0 %	74.7 %	68.2 %
Diabetes	33.7 %	33.8 %	32.5 %	35.4 %	29.0 %
Asthma	19.3 %	19.5 %	19.7 %	19.1 %	15.1 %
Arthritis	48.6 %	49.3 %	49.5 %	48.0 %	42.4 %
Risk Score	1.91	1.91	1.91	1.75	1.94

As can be seen, we were able to match 78 % of the total sample (Sample A) to the Medicare medical claims data available from the Centers for Medicare and Medicaid Services (CMS) using social security numbers, full names, and date of births (Sample B). Further, individuals who opted out of Medicare Plan B (ambulatory care coverage) or who chose a Medicare Advantage Plan for their 65^th^ or 66^th^ year (18 %) were excluded because we did not have access to their Medicare claims data (Sample C). Many of these individuals may well have still been working or on the insurance of a working spouse.

Because hourly workers (67 % of sample) tended to have longer tenures at this company than salaried workers, the data on these individuals was for a longer period of time and more complete. In addition, the vast majority of workers (hourly, or otherwise) were male (85 %). Thus, for many analyses, the data are limited to male, hourly workers (Sample D). In addition, people who died prior to age 70 (<4 %) were also excluded as outliers who likely had an abrupt change in health too close to the time of the Medicare transition (Sample E). Our sample does appear sicker than the greater sample; this is likely because we have more information on their health due to more complete claims data.

For analyses that consider whether there were discrete changes in medical diagnoses inferred from insurance claims, it was necessary to limit the individuals to those who worked at a plant that was not bought or sold during the observation period (Sample 1). For analyses for which continuous observation is necessary, the data are limited to individuals for whom continuous data are available in the years just prior to Medicare (Sample 2). In other sub-analyses, whether the plant was bought or sold was not relevant, but it was necessary to have other data, such as a risk score at age 61 (Sample 3), or BMI data from work-life (Sample 4), or lung function data from work-life (Sample 5).

### Primary variables of interest

#### Retirement

We considered an individual formerly engaged in hourly work as likely “retired” if he left employment after age 55, when basic retirement benefits became available for hourly workers. The median retirement age using this definition was 62, the age at which most workers were eligible for full benefits, and 75 % of hourly workers had left employment by age 64 [17]. It is, of course, possible that some of these workers left their jobs in manufacturing but were not purely retired and engaged in paid work elsewhere at this time.

#### Medical diagnoses

Medical diagnoses utilizing either private health insurance claims or Medicare data was measured using procedure codes as well as the International Classification of Diseases Version 9 (ICD-9) codes. Hypertension (401–405), diabetes (250), asthma and chronic obstructive pulmonary disease (COPD) (490–493, 496), major depression (296, 309, 311), and arthritis (710–720) were inferred if the code appeared on two or more face-to-face outpatient procedures within one year or one inpatient hospitalization. Coronary heart disease (410–414) was inferred if the code appeared on two outpatient visits or one inpatient hospitalization lasting for more than 48 h [20].

#### Risk score

The scores used were generated by private insurance carriers [21–23]. Although risk scores were initially calculated by actuaries to forecast health expenditures in the subsequent year, the scores have been demonstrated to be a powerful tool for indicating overall health status when surveys and diagnostic tests are not available (e.g., [24–26]). Risk scores have a national median of 1. Distributional properties and applicability to health services research is discussed at length in Hamad et al. [25].

#### Biometric data

Most hourly workers underwent regular medical exams at work that included general health measures—e.g., weight, cholesterol levels, blood pressure—and exams related to occupational exposures, most commonly audiometry and lung function. The latter data were available electronically for all workers at all plants, and the evaluation was performed using identical equipment at each location with routine and standardized training of technicians [27]. The general health information was abstracted since 2003 from the largest 20 locations; these data were not available for workers at smaller locations or those who left work before the times of data abstraction, thus resulting in incomplete data. A BMI of 30 or higher was considered “obese”. An abnormal lung function measurement is a Forced Expiatory Volume at one second (FEV1) and their Forced Vital Capacity (FVC) ratio of less than 0.7, was considered an indication asthma or COPD.

### Data analysis

This paper primarily relies on descriptive analyses. Concordance of claims using work and Medicare diagnoses are presented in terms of the proportion of “caught” diagnoses, or work diagnoses affirmed by a repeat diagnosis on Medicare. In addition, the precision and reliability of pre and post Medicare diagnoses are measured using kappa statistics, with common interpretations embedded [28], specifically, agreement labeled as poor (0.0–0.19), fair (0.20–0.39), moderate (0.40–0.59), substantial (0.60 –0.79), or near perfect (0.80–1).

Next, Ordinary Least Squares (OLS) regressions are used to examine whether there are any discontinuities in health consumption and diagnoses. This is done to determine whether individuals consume more or less medical care when transitioning onto Medicare from relatively generous private insurance. These data are repeated cross-sections, and therefore controlling for static individual characteristics is possible and necessary and conceptually essential. Specifically, person-level dummy variables were included in these models to account for static, individual characteristics. Minimal controls are included and results are not stratified by population because our interest was in the relationship between health consumption under Medicare and private insurance rather than moderators and mediators of such differences.

In addition, data from pre-Medicare life is used to predict outcomes for individuals. The results from these logistic regressions models are presented as Odds Ratios (ORs), or the odds of developing a disease given a specific characteristic or exposure divided by the odds of developing a disease in its absence. A score over one indicates that the variable of interest is associated with increased risk for this disease. Again, by using prior claims, static individual-level characteristics are effectively controlled for, but otherwise, minimal controls are included. The goal is not to assess causality but to demonstrate predictive power and potential. Two primary pre-Medicare individual-level characteristics are considered, as discussed below.

First, risk scores are examined for the work-life period. Specifically, we determined whether the risk score from when an individual was 61 predicts diagnosis of each chronic condition between ages 65–66 for individuals who did not already have this diagnosis at age 61. Risk score at age 61 was chosen because that is when we have access to the maximum number of insured individuals and it is prior to a large wave of leaving employment that occurred at age 62. Our analysis indicates that previous data on health claims can be used to bolster understanding of health for Medicare beneficiaries. Second, biometric data are utilized; specifically, data on lung function and BMI that were gathered at the jobsite. Our analysis demonstrates how prior information on risk can be informative regarding health later in life. These biometric data were gathered as part of plant-specific occupational health screening efforts. As such, individuals’ measurements were taken when workers were at different ages.

## Ethics approval

The Stanford University Institutional Review Board approved this study protocol, invoking the epidemiologic exemption waiving the requirement for individual consent.

## Results

### Concordance of medicare and work-life claims data

**Fig. 1:**
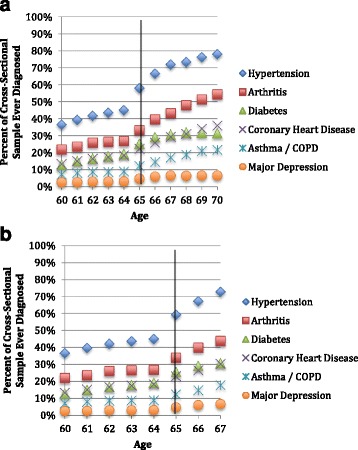
Cross-sectional prevalence since 1996, by age and Sample. **a** Male hourly workers (Sample 1). **b**: Male hourly workers with continuously observed coverage (Sample 2)

Figure 1 presents the cross-sectional prevalence of diseases by age for Sample 1. Note that the rates of illness are higher than a comparable US population. For example: the hypertension rate among US adult men ages 55–64 is 54 % (compared with 58 % at age 65 in this sample) [29]; the diabetes rate among US adults ages 45–79 is 13–14 % [30] (compared with 25 % at age 65 in this sample); and the COPD rate among US adults ages 55–79 is 10 % [31] (compared with 12 % in this sample). However, these data are consistent with biometric measures on this population.

Figure 1 appears to show an increase in the cross-sectional prevalence of each of the chronic diseases studied as individuals transitioned to Medicare. Of course, some diseases have an increase in prevalence with age; however, these increases should be smooth and a discrete jump is likely a result of idiosyncrasies within these two datasets. This may be due in part to sample selection—not all individuals were observed continuously. As seen in Fig. 1b, limiting the sample to those individuals who were observed continuously as they transitioned on to Medicare (Sample 2) attenuated but did not altogether remove this visually apparent spike.

Table 2 displays the concordance of Medicare versus earlier claims for Sample 2 to investigate why there appears to be an increase in diagnoses as individuals transition onto Medicare. As can be seen, all diseases do indeed appear to have had a higher cross-sectional prevalence at ages 65–66 using Medicare data (in particular, hypertension and arthritis) than in pre-Medicare private insurance. There was also a wide variation in whether a diagnosis from one’s working years was observed (or “confirmed”) in the initial two years of Medicare coverage. Chronic diseases such as hypertension and diabetes, which are typically permanent once diagnosed, had apparently highly confirmatory data. Episodic manifestations of chronic disease, such as arthritis and depression, were more likely to remain unconfirmed in the initial Medicare years. The most consistent diagnoses between Medicare and prior insurance (as measured by the kappa statistic) are for heart disease and diabetes, which are likely to have less ambiguous onset dates and will likely be a permanent feature of patients’ medical records.

**Table 2:** Concordance in diagnoses for Medicare and work-life claims (Sample 2)

	Hypertension	Diabetes	Heart Disease	Arthritis	Asthma	Depression
Dxs Prior to Age 65	44 %	19 %	18 %	26 %	9 %	3 %
Using 2 ICD-9 Diagnosis Codes						
Prior Dxs Confirmed at 65	68 %	78 %	57 %	29 %	39 %	11 %
Prior Dxs Confirmed by 66	81 %	88 %	68 %	41 %	45 %	20 %
New Dxs Seen at 65–66	22 %	10 %	8 %	14 %	6 %	3 %
Kappa age 64 and 65–66	0.57	0.36	0.65	0.32	0.18	0.21
Kappa rating	Moderate	Fair	Substantial	Fair	Slight	Fair
Using all ICD-9 Diagnosis Codes						
Prior Dxs Confirmed at 65	80 %	89 %	67 %	38 %	47 %	20 %
Prior Dxs Confirmed by 66	89 %	94 %	78 %	52 %	58 %	34 %
New Dxs Seen at 65–66	27 %	11 %	10 %	20 %	9 %	5 %
Kappa age 64 and 65–66	0.52	0.35	0.61	0.35	0.15	0.21
Kappa rating	80 %	89 %	67 %	38 %	47 %	20 %

All current and former hourly workers on Medicare with any health history were included in this sample (Sample 1). Sensitivity and specificity are considered using two ICD-9 codes as compared with all of the ICD-9 codes provided in the Medicare data

Note that Medicare data provides a large number of ICD-9 diagnosis codes (10 for each inpatient visit, 25 for each outpatient, and 12 for each visit covered under Part B), as compared with two in the private insurance we considered. We found that using all of the ICD-9 codes did not vastly increase the inferred new diagnoses but did capture more of the previously established diagnoses. Researchers who are working with Medicare data alone will be better able to capture rates of chronic disease if they use all of the available ICD-9 codes. However, even all ICD-9 codes do not fully inform on medical history of episodic or curable diseases.

### Healthcare utilization during transition to medicare

Table 3 examines healthcare utilization as individuals transition on to Medicare for Sample 1. These analyses are performed on repeated cross-section data. Both OLS and Poisson models are presented. Because there are repeat measures for individuals, a set of dummies for individuals is necessary. These controls remove variation caused by static individual characteristics (e.g., some people go to the doctor more often than others). Otherwise, the first models (OLS 1 and Poisson 1) are completely unadjusted. As can be seen, modeling the data in this way makes it appear that there is a large increase in medical consumption (both inpatient and outpatient visits) when individuals start obtaining Medicare coverage. It is worth noting that over 97 % of this population had a face-to-face outpatient visit within the first two years of Medicare coverage.

**Table 3:** Determinants of inpatient and outpatient visits (Sample 1)

a: Inpatient visits						
Inpatient	OLS 1	OLS 2	OLS 3	Poisson 1	Poisson 2	Poisson 3
Over 65yo	0.0500***	−0.0109	−0.00444	0.485***	−0.0679	−0.0262
	[0.00904]	[0.0202]	[0.0203]	[0.0667]	[0.151]	[0.150]
Retired			0.0559***			0.563***
			[0.0173]			[0.133]
Controls	--	+ Age	+ Age	--	+ Age	+ Age
b: Outpatient visits						
Outpatient	OLS 1	OLS 2	OLS 3	Poisson 1	Poisson 2	Poisson 3
Over 65yo	0.723***	0.0636	0.183	0.0341	0.0341	0.0494*
	[0.0981]	[0.149]	[0.0203]	[0.0279]	[0.0279]	[0.0281]
Retired			0.0559***			0.170***
			[0.0173]			[0.0371]
Controls	--	+ Age	+ Age	--	+ Age	+ Age

***p < 0.01, **p < 0.05, *p < 0.1

Three OLS models and three Poisson models are presented for each of the two outcome variables (inpatient and outpatient visits). These analyses are performed on repeated cross-section data. Because there are repeat measures for individuals, a set of dummies for individuals is necessary. These controls also remove variation caused by static individual characteristics (e.g., some people go to the doctor more often than others). In addition, standard errors are clustered by individual to account for serial correlation. Otherwise, model 1 is completely unadjusted. The model 2 includes an age polynomial, because there are increases in medical care consumption that occur as individuals age. Model 3 includes a measure of whether the individual retired

In the next set of models (OLS 2 and Poisson 2), age was modeled flexibly with a vector of polynomials that accounts for smooth increases in healthcare consumption due to age; the “over 65” dummy variable captures any discontinuous increases in visits at age 65, which may have been caused by the change in insurance coverage. We found no differences in inpatient visits as a function of turning 65. The majority of the sample (88 %) did not move to a different state after retiring and the majority of individuals, and likely continued to visit the same medical providers in Medicare as they did while covered by private insurance.

As can be seen in the OLS 3 and Poisson 3 models in Table 3, although the *transition* to Medicare was not associated with increased healthcare acute care utilization, *retirement* did predict additional inpatient and outpatient visits in this sample. This may indicate poorer health status among former workers. It is also possible that some of the increase in outpatient visits was caused by lower opportunity costs to seeking medical care among retirees. In Poisson 3, it does appear that transitioning to Medicare may be associated with slightly increased outpatient visits.

### Exploring earlier data to validate diagnostic inferences from medicare

**Fig. 2:**
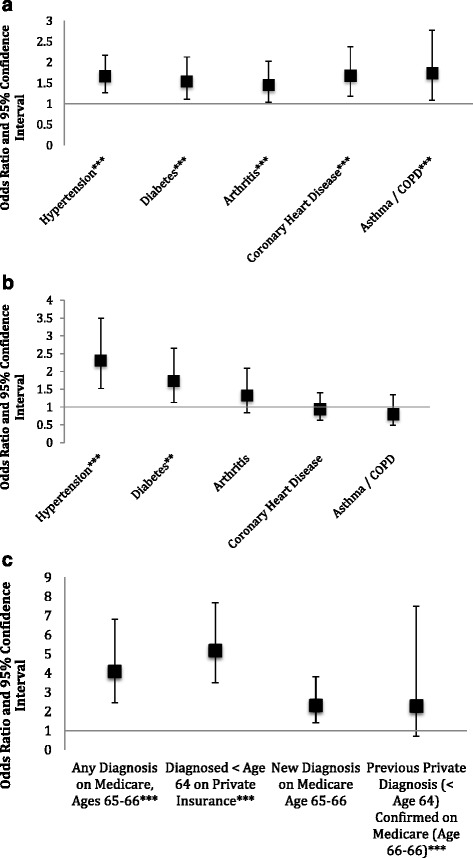
Early Medicare as a function of earlier data. **a**: High risk score at age 61 predicts new diagnoses (Sample 3). **b**: High previous BMI measurement predicts hypertension and diabetes (Sample 4). **c**: Abnormal lung function associated with asthma/COPD diagnosis (Sample 5)

For those individuals who had private insurance through work at age 61, a risk-score was available (Sample 3). This allowed us to evaluate whether health trajectories from pre-Medicare life predict outcomes for those over the age of 65. Figure 2 presents the ORs from logistic regressions, such that the coefficients are the odds of having a diagnosis of a specific new onset disease given a high risk score divided by the odds for individuals with low risk scores (a value of 1 indicates identical likelihood). As can be seen in Fig. 2a, having a risk score over the median for the subsample predicted new diagnoses of all of the chronic diseases considered. These results suggests that previous health trajectories can be informative about outcomes for individuals on Medicare, and may serve as a valuable tool for analyses that require controlling for prior health status.

For a subset of the population, biometrics including height, weight, and lung function were obtained at the jobsite. These data were integrated to explore how work-life administrative data can be used to provide a context for Medicare claims. Note that these measurements were taken at different ages, and therefore it was necessary to control for age and year of measurement. Plant of measurement was controlled for to account for any systematic differences between plants in gathering these data.

Figure 2b investigates the effect of having a BMI over 30 (43 % of the sample of individuals for whom we have BMI data, Sample 4). This figure presents the results of a set of logistic regressions on the added risk of having had high BMI measurement as compared to individuals who had always had BMI measurements under 30. As can be seen, high BMIs were associated with higher rates of diagnosis of hypertension and diabetes at ages 65–66. This supports the notion that Medicare claims represent true health outcomes. However, high BMI did not appear to be associated with increased rates of coronary heart disease. Because of the limited sample size of individuals for whom we have data on BMI, it was not possible to differentiate between previous and new diagnoses of the chronic diseases considered.

Figure 2c depicts findings on the effect of having had an abnormal lung function test (17 % of the sample of individuals for whom we have lung function data, Sample 5). Using logistic regression, individuals with an abnormal lung capacity result were compared to individuals with normal lung capacity results. In this sample, an abnormal result is associated with higher rates of COPD diagnoses on Medicare as well as on the private insurance. Further, those individuals who were diagnosed with lung disease using this biometric were found to be more likely to have a positive diagnosis on both private and public insurance. Finally, among those individuals with new diagnoses when they joined Medicare, lung function testing suggests that they were likely to have had previously measureable obstruction.

## Conclusions

This paper provides a first look into how private insurance data can be merged with Medicare data to provide a glimpse at health trajectories across the life course and after joining Medicare as primary insurer. This type of integration will be valuable to researchers trying to understand how health and risk trajectories emerge, investigating health outcomes of retirement, or comparing how policies and programs can alter the timing of retirement to improve long-term health outcomes.

Alone, both work-life claims and Medicare claims datasets have specific weaknesses. The data on employment-based claims files are often censored at age 65, when Medicare becomes primary for individuals who are not otherwise insured, or earlier for individuals who leave their workplaces. Thus, private healthcare claims provide a window into pre-Medicare health for still-employed individuals but little information on individuals who leave the labor market or who are over the age of 65. On the other hand, Medicare data provide no direct information about individuals’ health prior to age 65, and are not typically linkable to files containing more than superficial demographic, economic, behavioral, or environmental data on the covered individuals. Integration of both data sets allows for researchers to observe individuals both before and after age 65, and allows for Medicare data to be linked to administrative data on work-life characteristics and behaviors from individuals’ work-lives (e.g., tenure, absences, disciplinary actions, etc.).

### Limitations and future research

Our results do have some limitations. First, the specific results are not necessarily generalizable to other insured workforces. The sample studied here is a group of hourly workers in physically demanding jobs, and thus represent a very specific subset of the American population. Further, individuals in physically demanding jobs may retire younger than other workers [32]. Thus, industries with later retirement may have different health outcomes and trajectories. In other words, no one firm or industry will be fully generalizable, and any integration at the firm-level should be viewed as a unique look at a specific population’s health trajectory.

Another limitation of all linkages of this type is that some individuals non-randomly chose Medicare Advantage plans, the results presented are subject to some selection bias, as, prior to the passage of the Affordable Care Act, these plans were not required to report health claims. Newer regulations, stipulating that Medicare Advantage plans must report their data may mitigate this limitation, although interpretation of the mandate remains unresolved. If, ultimately, the claims-reporting requirement for Medicare Advantage plans is not enforced, this may limit the value of linkages like the one described in this paper. However, as the described cohort continues to age onto Medicare, it will soon be possible to examine *how* biased studies using Medicare data are likely to be; and how different the health profiles are of those who opt into a Medicare Advantage plan from others at age 64. Similarly, these linkages themselves are not perfect. Our algorithm linked 75 % of our potential sample to CMS data. It is not possible to know whether the unlinked individuals have different health trajectories than their linked counterparts.

Third, our data do not allow us to see whether individuals who leave their jobs are retired in a classic sense or whether they move on to other employment. Similarly, important context factors (e.g., whether one’s spouse is retired) are also not available. Linking these data with data from the Internal Revenue Service, a process that is currently underway, will allow us to see what proportion of these workers take on additional paid work after obtaining their pensions, and whether their spouse’s retirement status influences their decision to leave paid work in manufacturing and altogether.

Finally, this linkage was not able to examine the predictors of rare illnesses because the sample size was too small. As more of the cohort ages onto Medicare, our sample will grow larger and allow for evaluating risks for additional ailments, but it would take a very large sample to study very uncommon diseases.

### Final thoughts

By merging data from work-life with Medicare, it is possible to explore later-life health trajectories as a function of previous behaviors and exposures. Further, linking claims from one’s work-life allows for researchers to account for previous health and to better understand risk trajectories over time. Of particular value are data on work-life exposures, behaviors, and clinical data obtained during medical appointments. In addition merging these data with exposures during work-life would allow researchers in occupational health to utilize an outcome variable (i.e., health at age 65–66, as ascertained from Medicare claims) that is less biased by selection out of the workforce than private insurance claims, as well as more timely and relevant than cause and age of death. In sum, our paper provides a proof of concept for a valuable and promising innovation for research on health.

## Abbreviations

BMI: Body mass index
CHARLS: Chinese health and retirement survey
CMS: Centers for medicare and medicaid services
COBRA health insurance: Consolidated omnibus budget reconciliation act—refers to temporary group insurance available for purchase to individuals whose insurance might otherwise be terminated
COPD: Chronic obstructive pulmonary disease
FEV1: Forced expiatory volume at one second (FEV1)—a measure of lung function
FVC: Forced vital capacity—a measure of lunch function
HRS: The health and retirement survey
ICD-9: International classification of diseases version 9
NDI: National death index
OLS: Ordinary least squares
OR: Odds ratio
SHARE: Survey of health and retirement in europe
